# The return of the functional morphologists

**DOI:** 10.1007/s00709-022-01812-8

**Published:** 2022-10-08

**Authors:** Peter Nick

**Affiliations:** Botanical Institute, Karlsruhe Institute of Technology, Karlsruhe, Germany

Cell biology is a scientific discipline dealing with form in one way or the other. This remains true, albeit the molecularisation of biology has also changed the way, how cell biology is understood nowadays: The micropipette has often replaced the microscope. But, still, cell biology is more than finding out, which gene is activated or responsible for the process of interest. Cell biologists always ask, where things happen, in which tissue, in which cell, in which organelle. However, the description of the whereabouts is not an end by itself — to become fruitful, the “where” should remain integrated into the “what for”, the attempt to explain the function of the process. Form and function are two sides of the coin and must always be seen together, or, to put it in a *bonmot* by Vogel and Wainwright (1969), “Structure without function is a corpse, function without a structure is a ghost”.

But who is ruler and who is ruled? Does form follow function or does function follow form? Architects and designers tend to the first answer, following a seminal essay by Sullivan (1896), who, interestingly, used examples from biology to make his point “Whether it be the sweeping eagle in his flight, or the open apple-blossom, the toiling work-horse, the blithe swan, the branching oak, the winding stream at its base, the drifting clouds, over all the coursing sun, form ever follows function, and this is the law. Where function does not change, form does not change.” Generations of biologists have been taught that this holds also true for evolution, because change is driven by adaptation increasing “fitness”. Already, the wording reflects the idea that living is something fluid, which will “fit into” a space that is provided by the environment. The counterposition, namely that function follows form did never attain the same reachout. In their famous “Spandrels of San Marco” essay, Gould and Lewontin (1979) have impressively laid down that not every change of form can be derived from a function, but that form by itself can be a driver — the spandrels of San Marco were not made *in order* to provide the frame for the images inserted in them but were simply needed for the statics of the building. In a similar way, biological form can either enable or prevent the evolutionary development of a function. This thought can be used as foundation of an entire discipline — functional morphology, which from the 1980s became quite influential, especially in zoology (Wainwright 1988). While the study of biological form to access function has acquired momentum for technical applications, giving rise to bio-inspired engineering, often referred to as biomimetics or bionics (for a recent insightful review, see Speck and Speck 2021), it seems to play only a marginal role for current evolutionary theory. This is astonishing since the advances in functional genetics provide powerful tools to rephrase the question and link form with the ensuing function. Actually, this would be a cell-biological project. It requires a thorough description of the morphological details, the integration of gene expression and function with morphology, and an evolutionary perspective based on comparisons. Three contributions to the current issue highlight these individual ingredients for a new form of functional morphology:

The work by Bogolyubov et al. (2022) deals with very peculiar structures discovered by TEM in the nuclei of *Pelomyxes*, an exotic genus of protists that behave as ameba, while harbouring residual flagella, and lacking mitochondria and a Golgi apparatus. These intranuclear bodies are of almost geometric precision and contain parallel cords of electro-dense material. The authors baptise them glomerulosomes and very carefully delineate them from other types of intranuclear structures that have been described during the last decades. While at first site, these structures might appear linked with the nucleolus, the authors show that they are spatially separate. In order to get access to the potential functions of these glomerulosomes, they use a comparative approach by searching them in different species of this exotic genus. Interestingly, only four of the fifteen known *Pelomyxes* species harbour glomerulosomes, all deriving from a stagnant pond since a cultivation system for these creatures has not been developed yet. During the delineation of glomerulosomes based on often neglected old literature, they come across a similar structure observed in ciliates after treatment with actinomycin D (Bohatier 1977), which indicates that the presence of glomerulosomes in *Pelomyxes* might be linked with changes in the initiation of transcription in this ancient or strongly derived eukaryote.

The contribution by Weryszko-Chmielewska et al. (2022) adopts a classical functional morphology approach to understand nectaries in *Chaenomeles japonica*, a Rosaceous shrub, which is ecologically valuable, because it is very attractive for bees. The release of nectar is far from trivial, because sugar has to be secreted out from the cell and is then prone to become prey of fungi and bacteria, including pathogens that would endanger the precious object of attraction, the generative apparatus. Nature has, therefore, invented numerous tricks to both reward the pollinator while retaining their gift from undesired hitchhikers. Using a combination of Scanning and Transmission Electron Microscopy, classical histochemistry, and integration of functional data, the authors can show that secretion through stomata does not play a role here, but that epidermal cells do the job and that the curious striated structures of their cuticles help to keep the nectar that is released through pores and ruptures in the cuticle from drying, such that the pollinator will find an attractive reward. Interestingly, these epidermals cells accumulate tannins in their vacuoles, a feature that had also been reported earlier for *Viburnus* (Konarska 2017) and represents the morphological manifestation of the antifungal barrier that, in one form or the other, must also be a necessary component of nectar reward. Thus, an otherwise mysterious structure (the striated surface of the cuticle) can be neatly explained if one integrates morphology into a functional context. This explanation is of the *causa finalis* type, the question, by which mechanism these secretory striae develop, provides research questions for the future that are interesting beyond the pollination system of this shrub.

Also, the study by Piao et al. (2022) in the current issue deals with flower morphology but draws a link with developmental regulators. Their plant of interest, *Chelone glabra*, is a relative of *Antirrhinum*, a classical model for developmental genetics of flower morphology, and is of interest for studies in polyploid evolution. The surface of its anthers is densely covered with hairs, which are important to link the four stamina into a mechanically linked androeceum that very efficiently can deposit the pollen on anyone, who tries to get into the corolla (Cooperrider 1967). The authors tested the hypothesis that in this species, a regulator of trichome development should be upregulated, which led them to search for homologues of known regulators of trichome fate in model plants such as thale cress or cotton and clone out a homologuous gene from *C. glabra*. In fact, this homologue, *CgMYB4*, was upregulated in stamens. To address its developmental function, the authors first generated hairy roots, where *CgMYB4* was either overexpressed or silenced and observed that root hair density was responding as expected. Eventually, they managed to get *C. glabra* plants either overexpressing *CgMYB4* or downmodulating this gene by RNai. The overexpressors had more pronounced hairs, the silenced line less. When the histology was investigated in more details, the overexpressors were observed to produce conical cells in their epidermis, and more elongate cells in the subtending cell tier. This indicates that this transcription factor does not act by changing the patterning, but rather supports the manifestation of this pattern by promoting axis elongation.

All three contributions address the link between form and function, but they approach the study of morphological detail from different viewpoints. The glomerulosomes in *Pelomyxes* are discussed from an evolutionary perspective, the nectaries in *Chaenomeles japonica* are understood with respect to their function, nectar reward for pollinators, but simultaneously keep this reward from drying and attracting microbes, and the trichomes on the *Chelone glabra* anthers are investigated in the context of developmental genetics. The multitude of these accessory perspectives that are linked with the description of the structures themselves, leads to an important conclusion. We will never be able to disentangle the link between form and function by studying morphology alone. We need to integrate quite different scientific disciplines to arrive at a real understanding. This means also that cell biology cannot constrain itself to being the discipline dealing with form, it must widen up to integrate knowledge from other disciplines in order to be fruitful. The microscope and the micropipet are not mutually exclusive, but complementary.
